# Screening of Indigenous Oxalate Degrading Lactic Acid Bacteria from Human Faeces and South Indian Fermented Foods: Assessment of Probiotic Potential

**DOI:** 10.1155/2014/648059

**Published:** 2014-02-11

**Authors:** Sivasamy Gomathi, Ponnusamy Sasikumar, Kolandaswamy Anbazhagan, Sundaresan Sasikumar, Murugan Kavitha, M. S. Selvi, Govindan Sadasivam Selvam

**Affiliations:** ^1^Department of Biochemistry, Centre for Advanced Studies in Organismal and Functional Genomics, School of Biological Sciences, Madurai Kamaraj University (University with Potential for Excellence), Madurai 625 021, India; ^2^INSERM-U844, Hopital St. Eloi, Institut des Neuroscience de Montpellier Building, 34091 Montpellier, France

## Abstract

Lactic acid bacteria (LAB) have the potential to degrade intestinal oxalate and this is increasingly being studied as a promising probiotic solution to manage kidney stone disease. In this study, oxalate degrading LAB were isolated from human faeces and south Indian fermented foods, subsequently assessed for potential probiotic property *in vitro* and *in vivo*. Based on preliminary characteristics, 251 out of 673 bacterial isolates were identified as LAB. A total of 17 strains were found to degrade oxalate significantly between 40.38% and 62.90% and were subjected to acid and bile tolerance test. Among them, nine strains exhibited considerable tolerance up to pH 3.0 and at 0.3% bile. These were identified as *Lactobacillus fermentum* and *Lactobacillus salivarius* using 16S rDNA sequencing. Three strains, *Lactobacillus fermentum* TY5, *Lactobacillus fermentum* AB1, and *Lactobacillus salivarius* AB11, exhibited good adhesion to HT-29 cells and strong antimicrobial activity. They also conferred resistance to kanamycin, rifampicin, and ampicillin, but were sensitive to chloramphenicol and erythromycin. The faecal recovery rate of these strains was observed as 15.16% (TY5), 6.71% (AB1), and 9.3% (AB11) which indicates the colonization ability. In conclusion, three efficient oxalate degrading LAB were identified and their safety assessments suggest that they may serve as good probiotic candidates for preventing hyperoxaluria.

## 1. Introduction

Oxalate is a highly oxidized toxic substance that is widely distributed in nature. Some of food stuffs, particularly vegetables and cereals, contain high amounts of oxalic acid and can result in a significant increase in urinary oxalate excretion [1]. An increased oxalate intake and intestinal absorption may lead to hyperoxaluria, a predominant risk factor for calcium oxalate stone disease [2] which is characterized by a high frequency of recurrence. This also causes a range of deleterious clinical outcomes including urolithiasis, renal failure, cardiomyopathy, cardiac misconductance, and death in humans [3]. Recurrent stone formation is still common and the lifetime recurrence rate is likely to be 50%. Currently, existing invasive therapeutic strategies are ineffective to eradicate the stones completely causing recurrence [4]. Dietary restriction may not be a reliable approach to prevent recurrent stones as this may lead to nutritional deficiency. Humans lack the enzymes needed to metabolize oxalate. Hence, an effective prophylactic treatment is essential to overcome recurrent stone formation. Recent studies are focused on developing intestinal oxalate degrading bacteria as an appropriate probiotics solution to prevent kidney stone disease.

Probiotics are being abundantly used as preventive therapeutic agent for several diseases [5]. Probiotics are defined as live microorganisms which, when administered in adequate amounts, confer a health benefit on the host [6]. It can be implicated in stabilizing gut microbiota and enhancement of immune response and act as competitor against enteric pathogens [7]. Several studies on probiotic bacterial treatments have demonstrated promising results in ameliorating diseases including inflammatory bowel disease, irritable bowel syndrome, pouchitis, and acute infantile or antibiotic-associated diarrhea [8]. Numerous studies have documented that gut microbes maintain the oxalate homeostasis via utilizing the intestinal oxalate, while reducing the urinary oxalate excretion [9, 10]. *Oxalobacter formigenes (O. formigenes)* is an oxalate degrading bacterium, which uses intestinal oxalate as a sole source of carbon in order to regulate the oxalate homeostasis. However, its probiotic use has been limited due to fastidious nutrient requirements, less colonization ability, and specialized oxalotrophic nature. Lactic acid bacteria (LAB) are vital residents of human intestinal ecosystem and have been extensively used as probiotics owing to their health promoting benefits to the host [7]. Studies have confirmed the correlation between oral administration of *Lactobacillus* or *Bifidobacterium* species and their important role in luminal oxalate reduction, which decreased the risk of urinary oxalate excretion in humans and animals [2, 11–13]. Turroni et al. [14] reported a range of oxalate degrading lactobacilli from pharmaceutical and dairy products and found significant oxalate degradation in *Lactobacillus acidophilus* and *Lactobacillus gasseri.* However, the number of identified oxalate degrading bacterial species is limited and there is no report regarding the ability of oxalate degrading LAB from human gut microbiota. Alternatively, the use of recombinant LAB expressing heterogeneous oxalate degrading gene as a probiotic tool to control enteric hyperoxaluria was also suggested [15–17]. The present study is aimed to screen an efficient oxalate degrading LAB from human faeces and south Indian fermented foods and to evaluate the safety assessment of potential probiotic characteristics both *in vitro* and *in vivo*.

## 2. Materials and Methods

### 2.1. Sampling and Isolation of LAB

Human faecal samples were collected from thirty healthy individuals (mean age of 23–40) who had not taken antibiotics and probiotics at least for the past three months. Samples were collected in sterile container, kept in ice box, transported to laboratory within one hour, and processed immediately. South Indian traditional fermented foods used in this study were homemade preparations. Fresh curd, fermented appam batter (prepared by grinding the presoaked parboiled rice and dehulled black gram and allowed for natural fermentation for 12–24 h), and fermented wheat kali (paste prepared by slowly adding the wheat flour into boiling water and stirred continuously until correct consistency. This was made as balls, soaked in water, and allowed to ferment naturally) were used to isolate oxalate degrading LAB.

To isolate LAB, one gram of each faecal and fermented food sample was added separately to 9 mL of 1% peptone water and homogenized. Tenfold dilution was prepared with peptone water and appropriate dilutions of each sample were spread on de Man Rogosa Sharpe (MRS) (Himedia, Mumbai, India) agar plate and incubated at 37°C for 72 h. From each sample, 15–30 colonies were randomly selected and purified by streaking with MRS agar plates. Pure cultures were preliminarily characterized based on gram staining, catalase reaction, and clear zone formation in 0.5% of CaCO_3_ plate, glucose fermentation, and arginine hydrolysis [18]. Tentatively identified LAB isolates were stored at −80°C in MRS broth with 20% glycerol.

### 2.2. Determination of Oxalate Degrading Ability

The presumptive LAB was screened for oxalate utilization using agar well-diffusion method in calcium oxalate plate and was prepared as described by Allison et al. and Campieri et al. [9, 11]. Wells of 6 mm diameter were prepared in calcium oxalate plate and each well was inoculated with 0.1 mL of overnight culture and incubated at 37°C for 12 h. The oxalate utilizing bacteria can form clear zone around the well due to oxalate decomposition by the isolates. Zone diameter was measured and the isolates displaying 10 mm of zone were subjected to quantitative determination of oxalate degradation. To examine their ability to degrade soluble oxalate, the isolates were cultured in 5 mL of MRS broth supplemented with 10 mM of potassium oxalate (KOX) for 5 days and MRS broth without bacterial inoculum was used as control. Prior to oxalate determination, the control as well as bacterial culture was processed using method of Federici et al. [19]. The oxalate concentration from the supernatant was determined as described by Hodgkinson and Williams [20]. Consider

(1) (%)Oxalate  degradation=(Oxalate  concentration  in  KOX  control−Oxalate  concentration  in  supernatant)(Oxalate  concentration  in  KOX  control)×100.

### 2.3. Acid and Bile Tolerance Test

The acid tolerance of LAB isolates was evaluated in simulated gastric juices and bile salt tolerance of LAB isolates was determined at 0.3% bile concentration using the modified method of Wang et al. [21]. Cell suspensions containing ~1 × 10^7^–10^9^ cells were inoculated into MRS broth supplemented with or without 0.3% (w/v) of bile. After 12 h incubation, 0.1 mL of each culture was serially diluted with 1% of PBS and spread on MRS agar plate. The plates were incubated aerobically at 37°C for 72 h. The viable cells were counted using plate count method and expressed as mean log CFU/mL. The survival (%) of the bacteria was calculated as follows:

(2) $$\begin{array}{c} \%\;\;\text{Survival}=\left(\frac{\text{log number of viable cells survived (CFU/mL)}}{\text{log number of initial viable cells inoculated (CFU/mL)}}\right)\times 100. \end{array}$$

### 2.4. DNA Extraction, PCR Amplification, and Sequencing of 16S rDNA

The bacterial genomic DNA was extracted as described elsewhere [22]. All fine chemicals and primers were procured from Sigma Aldrich (USA). PCR amplification of 16S rDNA was carried out by using primers Lab-0677 (CAC CGCTACACATGGAG) and Bact17 (AGAGTTTGATCATGGCTCAG) which produce 700 bp amplicon [23]. PCR reaction was performed in GeneAmp PCR system 2700 (Applied Biosystem, USA). The cyclic program consisted of an initial denaturation at 94°C for 5 min; 35 cycles of 94°C for 40 s, 56°C for 45 s, and 72°C for 1 min were followed by a final extension period at 72°C for 7 min. The amplified PCR products were purified using Gene Elute PCR Cleanup Kit (Sigma Aldrich, USA) and sequenced commercially (Eurofins Genomics India Pvt., Ltd., India). The 16S rDNA sequences were compared with the sequence in GenBank Public database using BLAST software (http://blast.ncbi.nlm.nih.gov/Blast.cgi).

### 2.5. Antimicrobial Activity Assay

The antimicrobial activity was determined as described by Wang et al. (2010) against *Escherichia coli* ATCC 25922 (*E. coli*), *Staphylococcus aureus* ATCC 6538 (*S. aureus*), *Pseudomonas aeruginosa* ATCC 27853 (*P. aeruginosa*), *Bacillus cereus NCIM 245 (B. cereus), Bacillus subtilis *ATCC 6633 (*B. subtilis*), and* Salmonella typhi *25 (*S. typhi*) which are the indicator strains.

### 2.6. Antibiotic Susceptibility Test

Antibiotic susceptibility test was carried out for nine isolates as described by Bauer et al. [24]. The concentrations of antibiotics used per disc were 10 *μ*g of ampicillin, 30 *μ*g of tetracycline, 30 *μ*g of kanamycin, 15 *μ*g of erythromycin, 30 *μ*g of chloramphenicol, and 5 *μ*g of rifampicin. The zone of inhibition was measured and the results were expressed as susceptible (S), intermediate (I), and resistance (R) as per NCCLS standards [25].

### 2.7. *In Vitro* Adherence Assay

Adherence capacity of isolates was evaluated using HT-29 monolayer cells described by Verdenelli et al. [26] with some modifications. Briefly, cells were routinely grown in minimal essential medium (MEM) (Himedia, India) containing 2 mM L-glutamine, 1 mM sodium pyruvate, 1% nonessential amino acid, 1.5 g/L sodium bicarbonate, 10% fetal bovine serum, 50 U/mL penicillin, and 0.05 mg/mL streptomycin. To investigate the adhesion ability of isolates, HT-29 cells were seeded at 1.5 × 10^5^ cells per well in 24-well tissue culture plate and incubated at 37°C with 5% carbon dioxide for 24 h incubation followed by washing three times with phosphate buffered saline (PBS). Each bacterial culture was diluted up to 10^8^ cells/mL by MEM medium and inoculated into HT-29 monolayer cells. After 2 h of incubation, the monolayer was washed three times with 1 mL of PBS to remove nonadhered cells and lysed by the addition of 0.25 mL of 0.1% (v/v) Triton-X100 in PBS for 10 min at 37°C. The lysate was plated on MRS agar after a series of dilutions and incubated for 24 h to 48 h for bacterial enumeration. Adherence percentage was calculated by comparing the adhered cells to the total cells of bacterial suspension.

### 2.8. Growth Kinetic Analysis of Oxalate Degrading Bacteria

Kinetic analysis of growth and oxalate degradation of isolates was evaluated. The oxalate degrading strains were inoculated in MRS broth supplemented with 10 mM potassium oxalate and noninoculated broth was used as control. The growth was monitored by reading absorbance at 600 nm at 24 h time interval. The absorbance at A_600 nm_ versus time curve was plotted to reveal the growth kinetics. Similarly, the oxalate degrading ability was also determined for every 24 h. Quantifying oxalate in the growth medium was determined as previously described [20].

### 2.9. Intestinal Colonization Ability of Oxalate Degrading LAB Isolates in Rat Model

Based on oxalate degrading efficiency and adherence ability, three representative strains *Lactobacillus fermentum* TY5 (*L. fermentum* TY5), *Lactobacillus salivarius* AB11 (*L. salivarius* AB11), and *Lactobacillus fermentum* AB1 (*L. fermentum* AB1) were selected. The survivability and colonization ability of oxalate degrading LAB were assessed using a rifampicin-resistant spontaneous variant (Rif^R^) [27]. The Rif^R^ strains were tested for growth properties and oxalate degrading abilities compared to the original strains. The selected Rif^R^ strains were used to colonize the rat intestine by oral administration. Sixteen male albino Wistar rats with an average body weight of 150 g–180 g were used in the study. Food and water were provided *ad libitum* for the study period. The animal protocol was approved by the Internal Research and Review Board, Ethical Clearance, Biosafety and Animal Welfare Committee of Madurai Kamaraj University. Rats randomly assigned into Group 1 were given 1 mL of saline by esophageal gavage (control). Group 2 received 10^8^ cells/day of *L. fermentum *TY5. Group 3 received 10^8^ cells/day of *L. salivarius* AB11. Group 4 received 10^8^ cells/day of *L*. *fermentum* AB1. The above strains were administrated for one week, which was followed by a washout period of one week. The faecal pellets were collected prior to probiotic administration (day 0) and on days 3, 7 and 14.

The impact of probiotics on rat gut microbiota was evaluated using culture-dependent analysis in faecal samples. Rat faecal sample was processed in the same way as human faecal sample and suitable dilutions were plated in duplicate on selective plates as described by Bernbom et al. [28]. Reinforced clostridial agar (Himedia, India) was used to selectively grow members of clostridia family; MacConkey agar number 3 (Himedia, India) was used for total coliforms; *Lactobacillus* was cultured in MRS agar. Nutrient agar was used for total facultative aerobes (Himedia, India). Thioglycolate agar was used for total anaerobes (Himedia, India); finally MRS containing rifampicin 0.1 mg/mL was used to select Rif^R^ probiotics. The viable cells were counted and expressed as log CFU/g of faeces.

### 2.10. Statistical Analysis

The results were expressed as mean ± standard deviation (SD). Student's paired* t-*test was performed to compare mean oxalate degradation and to compare the tolerance (acid and bile) test, the viable counts were transformed to log_10_ values before statistical analysis. Student's unpaired *t-*test was used in a case of animal model to compare lactobacilli fed group versus control. A significant difference was accepted at *P* < 0.05. Statistical analysis was performed using XLSTAT (2013.4.04).

## 3. Results

### 3.1. Identification of Oxalate Degrading LAB

Screening of oxalate degrading bacteria from human faecal samples demonstrated the presence of significant population of oxalate degrading LAB in the human intestine. Among 30 individuals, 22 possessed LAB with oxalate degrading ability while eight individuals contained *Lactobacillus* without oxalate degrading property. Totally, 673 strains were isolated from human faeces and fermented foods (appam batter, wheat kali, and curd), among which 251 strains were LAB based on preliminary identification. All strains were Gram positive and catalase negative and able to form zone in 0.5% CaCO_3_ and to be positive for glucose fermentation. Ammonia production was observed in 93 strains. The presumptive LAB was examined for oxalate utilization using calcium oxalate plate. A total of 92 oxalate degrading strains were detected. Among them thirty-seven colonies showed clear zone above 10 mm, and they were further subjected to evaluation of oxalate degrading efficiency.  Table 1 shows the oxalate degrading ability of selected isolates. The isolates were able to grow and degrade oxalate in the presence of 10 mM potassium oxalate, but the degree of oxalate degradation was varied. Significant oxalate degradation was observed in seventeen strains, out of which ten strains utilized more than 50% of oxalate. In particular, the isolates AB11 and TY12 appeared to be active and showed the highest oxalate utilization.

### 3.2. Acid and Bile Tolerance

In order to choose potent LAB candidates for the use as probiotics, strains showing high degree of oxalate degradation were selected for probiotic assessment. All the tested isolates were viable at pH 3.0 for 3 h indicating that almost all strains were acid tolerant (Table 2). However, only eight strains exhibited significant survival rate (*P* < 0.05). The maximal survival rate was observed in isolates AM3 and ER5 with 89.5% and 94%, respectively. Four strains showed reduced viable cell count and the remaining strains showed the least survivability. When exposed to pH 2, the survival rate was strongly reduced in AM15, AM20, MSS39, and W21 and no viability was observed in TY14, PR3, PR45, and C2 strains, which suggested that these isolates were highly sensitive to highly acidic pH. The tolerance to bile at 0.3% showed survivability of oxalate degrading LAB (Table 3). Among the tested strains, six strains showed significant survival rate (TY12, AM3, ER5, MM39, AB11, and AB1) while other strains were sensitive to bile. Based on acid and bile tolerance survival rate, nine strains (TY5, TY12, AM3, ER5, MM39, MSS10, AM3, AB11, AB1, and AB8) were selected for further 16S rDNA identification and probiotic evaluation.

### 3.3. Identification of Isolates Using 16S rDNA Sequencing

The 16S rDNA sequence of isolates was subjected to BLAST program for analyzing sequence similarity at NCBI database. Based on 16S rDNA sequencing, the isolates were found to belong to five *L. fermentum*, two *W. confusa*, one *L. salivarius*, and one *W. cibaria* species (Table 4).

### 3.4. Antimicrobial Activity

Nine isolates selected on the basis of their acid and bile tolerance were subjected to further probiotic evaluation.  Table 5 shows antagonistic activity of LAB isolates against six pathogens with variable inhibitory effect. The selected strains exhibited antagonistic activity, but varied in their inhibitory effect against each indicator bacterium and inhibited both Gram-positive and Gram-negative pathogens. *P. aeruginosa *ATCC 27853 was strongly inhibited by TY5, TY12, and AM 3, while other strains showed moderate inhibition. Most of the isolates showed moderate inhibition fairly against *B. subtilis *ATCC 6633. Five strains exhibited strong inhibition against *B. cereus* NCIM 2458. AM3 and AB11 showed inhibition against *S. aureus* ATCC 6538. *S. typhi* 25 was more resistant to most lactobacilli isolates, except TY5 and MSS10 that showed weak inhibition. Faecal isolates showed better inhibition towards pathogens than food product isolates.

### 3.5. Antibiotic Test

The antibiotic susceptibility of LAB isolates is showed in Table 6. All strains were resistant to kanamycin and rifampicin (except AB8) and susceptible to chloramphenicol (except ER5). Seven strains were resistant to ampicillin and one strain was intermediate whereas two strains were sensitive. Most of the isolates were susceptible to erythromycin, two strains were intermediate, and two were resistant. Tetracycline resistance was observed in three isolates, four were intermediate, and two were susceptible.

### 3.6. Adhesive Property of Isolates

**Figure 1:**
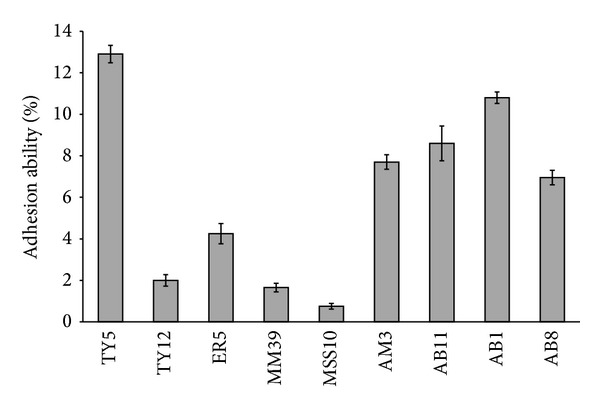
Adhesion ability of LAB isolates to HT-29 cells. The result was represented as mean ± SD of duplicates. The error bar indicates standard deviation.

The result of adherence ability of isolates is shown in Figure 1. The adherence ability of strains to HT-29 cells noticeably differed from each tested strain. *L. fermentum* TY5, a faecal isolate, was the most adhesive strain with 12.9% of bacteria bound to the HT-29 cells. Surprisingly, fermented food product isolate of  *L. fermentum* AB1 adhered with 10.8% to HT-29 cells. Other strains *L. salivarius* AB11, *L. fermentum* AM3, and *L. fermentum* AB8 exhibited moderate adhesion with 8.6%, 7.7%, and 6.9%, respectively.

### 3.7. Kinetic Analysis of Oxalate Degrading Ability Correlated with Glucose Consumption

**Figure 2:**
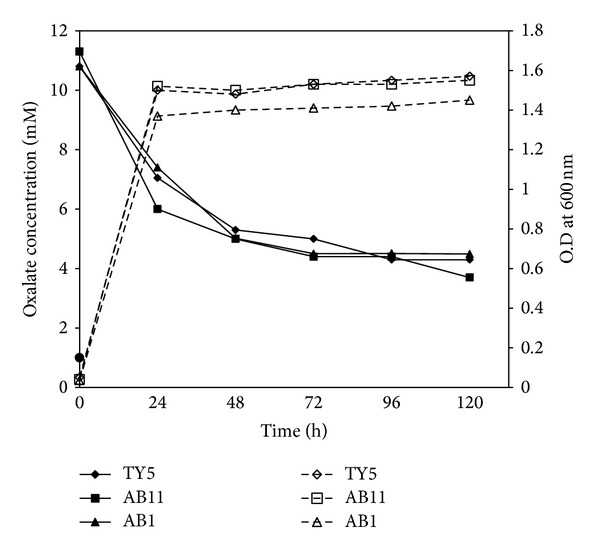
Kinetic analysis of oxalate degradation and growth curve of isolates in the presence of 10 mM KOX. Oxalate concentration present in the supernatant (straight line) was mostly consumed during 24 h growth (dotted line) corresponding to active growth phase of isolates.

To determine the impact of oxalate on growth, the growth kinetics and oxalate degrading ability were analyzed at 24 h time interval for 5 days. The oxalate degrading profile demonstrated that the majority of oxalate was degraded during 24 h, while gradual oxalate degradation rate was observed up to 72 h and it was sustained until 120 h (Figure 2). The degrading efficiency varied between isolates, but the pattern of the oxalate degradation was the same in all the isolates. The oxalate degrading activity was also examined in the absence of glucose with 10 mM KOX. In this condition, the isolates were unable to grow and utilize oxalate, implying the need for glucose to utilize oxalate.

### 3.8. Analysis of Intestinal Colonization Ability of LAB Isolates in Rat

**Figure 3:**
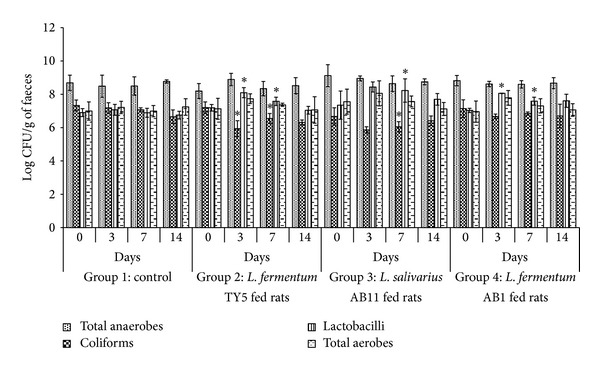
Analysis of faecal microbiota composition in rats before and after oral administration of selected *Lactobacillus*. Each rat administered ~10^8^ cells/day for one week. Viable counts of total anaerobes, coliforms, lactobacilli, and total aerobes expressed in terms of log CFU/g of faeces on days 0, 3, 7, and 14. Each value was represented as mean ± SD of log CFU/g wet faeces (*n* = four rats per group). *Significant difference in viable counts.

**Figure 4:**
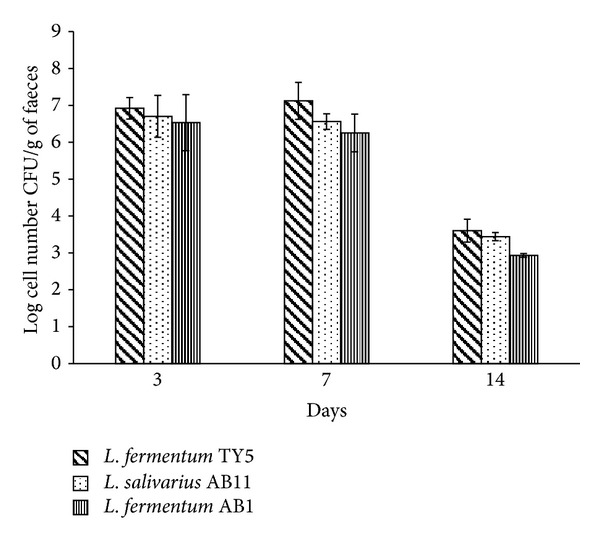
Recovery of Rif^R^ strains from rat faecal samples on days 3, 7, and 14. The data is represented as mean value of log CFU/g faeces. Rif^R^ strains were recovered on days 3 and 7 and decreased on washout period.

The intestinal colonizing ability and faecal microbiota after oral administration of LAB isolates were analyzed in rat model by conventional plate count method on selective agar.  Figure 3 shows the viable counts of microbial changes of faecal microbiota. None of the groups showed significant changes in total anaerobic and total aerobic counts. All groups showed significant increase in *Lactobacillus* species up to 7 days but it was reduced on washout period. *L. fermentum* TY5 and *L. salivarius* AB11 fed group showed significant reduction of coliforms and also showed considerable survival up to 14 days. The survivability of Rif^R^ oxalate degrading LAB was tested on 0.1 mg/mL Rif plate. Before probiotics administration, faecal sample was tested for presence of Rif^R^ colonies from lactobacilli fed group and control. None of the Rif plates showed Rif^R^ colonies, which confirmed the absence of Rif^R^ colonies. The Rif^R^ colonies were observed in faeces from lactobacilli fed rats on day 3 of administration which shows the gastric transit ability of consumed lactobacilli. On day 7, the faecal recovery rate was observed as 15.16%, 9.3%, and 6.71% in *L. fermentum* TY5, *L. salivarius* AB11, and *L. fermentum* AB1, respectively, and this was reduced on washout period (Figure 4). This represents that the consumed lactobacilli have the gastrointestinal transit and intestinal colonizing ability.

## 4. Discussion

In the present study, human faecal samples and traditional south Indian fermented food products were chosen in order to identify efficient oxalate degrading bacteria which could be used as probiotic supplements for their application in hyperoxaluria prevention.

Human intestinal isolates showed high degree of variability in oxalate degradation. Turroni et al. [14] found that *Lactobacillus acidophilus* and *Lactobacillus gasseri* showed significant oxalate degradation in 5 mM oxalate whereas other strains showed less oxalate consumption especially, *Lactobacillus salivarius* which showed 20% oxalate degrading ability. In this study, the maximum oxalate degradation was detected in isolates of *L. salivarius* AB11 (62.59%) and *L. fermentum* TY12 (58.3%) and five strains of *L. fermentum* sp. differed in their oxalate degrading ability. These results showed that oxalate degradation was both species and strain specific. Murphy et al. [27] also reported that oxalate utilization among probiotics *in vitro *was interspecies dependent. Among the identified oxalate degrading LAB strains, *Weissella confusa* and *Weissella cibaria* have the ability to degrade oxalate between 40 and 50%. Members of the genus *Weissella* have been isolated from various sources including human faeces, Korean kimchi, fresh vegetables, sugar cane, carrot juice, raw milk and sewage, milking machine slime, soil, fermented sausages, and meat products [29].

Seventeen efficient oxalate degrading strains were evaluated for acid and bile tolerance activity. Among the tested isolates, eight strains exhibit significant tolerance at pH 3 after 3 h incubation but the viability was reduced at low acidic condition. Previous studies have reported that most of the *Lactobacillus* sp. exhibited good survival at pH 3, with lower viability when exposed to pH 2 [21]. Resistance among *Weissella* sp. varied and the survivability rate was low at pH 2 [30]. Their findings support our result on acid tolerance of faecal and fermented food product LAB isolates. Several studies have demonstrated that the *L. fermentum* strains could survive at pH 2 and the outstanding survival rate was observed as around 96% at pH 3 [31, 32]. Similarly, in this study, *L. fermentum *AM3 strains showed 89.5% of survival rate in agreement with previous reports. Since LAB need to attain resistance to physiological bile concentration (0.3–0.5%) in the gastrointestinal tract, LAB isolates were evaluated for bile acid resistance. Five strains exhibited significant tolerance against 0.3% bile; *L. salivarius* AB11 and *W. cibaria* MM39 showed the highest bile tolerance activity similar to the findings observed in previous reports [30, 33].

Nine strains were selected on the basis of acid and bile tolerance for further study on antibacterial activity, one of the criteria in selection of probiotics, which is essential to protect host gastrointestinal tract from invading pathogens. This can be achieved by possible colonization of probiotics against pathogens [34]. Almost all the strains exhibited antagonistic activity against both Gram-positive and -negative pathogens. *L. fermentum* TY12 and AM3 and *L. salivarius* AB11 exhibited strong antagonistic activity against pathogens while other strains showed weak inhibition. *L. fermentum* TY12 and AM3 strains showed strong inhibition whereas *L. fermentum* AB1 and AB8 strains showed weak inhibition towards indicator strains this indicates the production of different antimicrobial substance by the selected isolates. The antagonistic activity of different lactobacilli strains varied towards pathogens due to strain specific nature [35] and differences in production of inhibitory compounds by lactobacilli spp. such as lactic acid, H_2_O_2_, and bacteriocin [36].

Safety assessment of probiotics including antibiotic resistance is an essential task in the selection of probiotics. *Lactobacillus* possessing any transferable resistance plasmid is believed to be a risk for human and animal use [37]. Intrinsic antibiotic resistant probiotics possibly will promote the patients with disturbed microbiota due to the administration of other antibiotics [38]. All nine strains were kanamycin and rifampicin resistant and susceptible to chloramphenicol that is in accordance with previous results [21]. Danielsen and Wind [39] found that transferable resistance gene such as chloramphenicol, erythromycin, and tetracycline may be present among LAB. However, three strains showed tetracycline resistance which is similar to previous report [21]. Several studies have proven that potential probiotics with atypical tetracycline resistance were conquered by plasmid curing which could have the susceptible phenotype [40]. In this study, most of the isolates were sensitive to chloramphenicol and erythromycin which revealed that the possibility of gene transfer to pathogen is less.

Adhesion and colonisation of probiotic bacteria in the gastrointestinal tract of the host are believed to be one of the essential features required for the delivery of their health benefits [41]. The adhesion capacity of strains was varied from 0.75 to 2.9% which showed strain specificity. This is consistent with earlier reports which suggested that adhesion to Caco-2 cells was found to be a discriminative parameter, showing marked variation among the strains independent of the species [42]. Among the tested strains, *L. fermentum* TY5 was the most adhesive strain which showed 12.9% adhesion to HT-29 cells similar to previous studies reported by Verdenelli et al. [26]. Fermented food strains also exhibited good adhesion to HT-29 while other isolates from faeces showed weak adhesion. This result is contrary to Wang et al. [43] who reported that adhesive *Lactobacillus* strains have host- residential characteristics. Wang et al. [21] also reported that fermented food isolate C06 exhibited strong adhesion than faecal isolate.

Lactobacilli play a pivotal role in maintaining the gut microbial balance and provide a barrier effect via the specific competitive action to pathogen for colonizing the intestinal mucosa [44]. The probiotic administration to healthy organism should not alter the naturally existing microbial balance in intestine. Otherwise, the subjects with imbalanced microbiota show adverse effect which was observed in case of antibiotic-associated colitis, infectious disease, and chronic pancreatitis [45, 46]. In this study, intestinal colonization ability and faecal microbiota changes were evaluated *in vivo* using rat model. *Lactobacillus* count was increased while coliform count was reduced significantly in two groups of rats. The microbial changes of rat intestine are similar to previous reports by Wang et al. [21] who reported significant increase in *Lactobacillus* and decrease in faecal coliform. Yang et al. [47] also observed the reduced faecal coliform counts due to appropriate beneficial role of *Lactobacillus* and *Bifidobacterium* proliferation and the inhibited invasion of pathogens in rat gut. The Rif^R^ colonies that were present in rat faeces after probiotic administration revealed that the strains were viable in gastric juice of stomach and gut transit. The good recovery rate in faeces suggested good colonization ability of strains. The recovery of Rif^R^ colonies was in accordance with previous report by Oozeer et al. [48] that suggested that multiplication of *Lactobacillus* in the colon leads to high faecal survival rate.

## 5. Conclusion

The present study showed oxalate degrading ability and probiotic assessment of human faeces and fermented food isolates. Three strains of LAB, *L. fermentum* AB1, *L. fermentum* TY5, and *L. salivarius *AB11, have efficient oxalate degradation together with good adherence to HT-29 cells, tolerance to acid and bile, strong inhibition against pathogens, and absence of transferable antibiotic resistance, which indicates that these strains show satisfactory properties for probiotic applications. Additionally, these strains survived well during gastrointestinal transit and reduced the coliform counts in faeces with good faecal recovery rate in rat model suggesting that these interesting probiotic properties make them as potentially good probiotic candidates which could be utilized for prophylaxis of calcium oxalate stone disease.

## Figures and Tables

**Table 1:** Determination of oxalate degrading ability of LAB isolates.

Source	Isolates	Oxalate concentration in supernatant (mM)	Oxalate degradation (%)
Human faeces	TY2	5.4 ± 0.72	48.07
	TY5	4.7 ± 1.17^a^	54.8
	TY12	4.33 ± 0.8^a^	58.3
	TY14	5.5 ± 1.18^a^	47
	AM2	5.83 ± 0.57	43.94
	AM3	4.5 ± 1.05^a^	56.7
	AM12	6.4 ± 1.23	38.46
	AM15	5.66 ± 0.59^a^	46.15
	AM19	5.4 ± 1.2	48
	AM20	4.5 ± 0.96^a^	56.73
	AM48	7.03 ± 1.02	32.35
	PR3	5.5 ± 0.96^a^	47.11
	PR14	6.2 ± 0.7^a^	40.38
	PR16	6.3 ± 1.77	39.42
	PR36	6.67 ± 0.65	35.86
	PR45	6.16 ± 0.77^a^	40.77
	PR56	5.9 ± 0.75	43.27
	PR63	7.21 ± 1.19	30.67
	TH14	6.6 ± 1.19	36.54
	ER1	6.3 ± 0.6	39.42
	ER3	7.3 ± 1.4	29.76
	ER5	5.83 ± 0.6^a^	43.94
	ER48	8.3 ± 0.75	20.19
	MM3	8.5 ± 0.83	18.2
	MM8	6.3 ± 0.5	39.68
	MM38	6.23 ± 1.07	40
	MM39	4.7 ± 0.66^a^	54.8
	MM40	6.7 ± 1.25	35.57
	MM42	6.9 ± 0.60	33.65
	MSS10	4.93 ± 0.57^a^	52.8
	AB11	3.9 ± 0.4^a^	62.49

South Indian fermented food	C2	5.53 ± 0.61^a^	46.82
	C14s	6.83 ± 0.45	34.32
	AB1	4.6 ± 0.5^a^	55.76
	AB8	4.22 ± 0.66^a^	59.42
	AB10	6.64 ± 0.54	36.15
	W21	6.08 ± 0.3^a^	41.5
	KOX Control	10.4 ± 1.00	

Values are expressed as mean ± SD from three trials. ^a^Significant difference (*P* < 0.05) in oxalate degradation compared with KOX control using Student's paired* t*-test.

**Table 2:** Survivability of LAB isolates at acidic condition.

Isolates	Initial mean count^1^	pH 2	Survival rate (%)	pH 3	Survival rate (%)
TY5	7.25 ± 0.27	4.63 ± 0.15^a^	63.8	5.74 ± 0.36^a^	79.17
TY12	6.67 ± 0.85	4.59 ± 0.84^a^	68.89	5.43 ± 0.88^a^	81.48
TY14	6.5 ± 0	—	—	3.96 ± 0.28	66
AM3	7.29 ± 0.20	4.21 ± 0.97	57.75	6.52 ± 0.1	89.5
AM15	8.41 ± 0.77	3.35 ± 0.07	39.8	4.99 ± 0.76	59.3
AM20	7.27 ± 0.05	3.76 ± 0.25	37.96	5.4 ± 0.5	74.27
PR3	6.41 ± 0.05	—	—	3.66 ± 0.31	57.09
PR14	6.51 ± 0.15	3.41 ± 0.07	52.38	4.46 ± 0.45	68.5
PR45	6.81 ± 0.06	—	—	4 ± 0.12^a^	54
ER5	6.8 ± 0.19	4.06 ± 0.36	67.7	6.37 ± 0.25^a^	94
MM39	7.62 ± 1.07	2.12 ± 0.1	34.2	4.83 ± 0.04	63.38
MSS10	7.22 ± 0.07	3.44 ± 0.08	60.3	5.88 ± 0.14	81.44
AB11	7.24 ± 0.09	6.02 ± 0.16^a^	67.44	6.73 ± 0.11^a^	88.7
C2	7.57 ± 0.45	—	—	3.06 ± 0.33	40.42
AB1	8.65 ± 0.07	6.71 ± 0.17	61.5	6.88 ± 0.18^a^	79.53
AB8	8.35 ± 0.26	7.12 ± 0.07	72.72	7.05 ± 0.09^a^	84.43
W21	7.88 ± 0.36	2.64 ± 0.26	33.50	4.71 ± 0.28^a^	43.11

^1^Viable counts were transformed to log CFU/mL and expressed as mean ± SD in three experiments. ^a^Significant difference (*P* < 0.05) at log_10 _measurements after 3 h incubation compared to the initial count. (—) No growth.

**Table 3:** Survivability of LAB isolates at 0.3% bile.

Isolates	Initial mean count^1^	0.3% bile	
		24 h	Survival rate (%)
TY5	8.8 ± 0.77	7.02 ± 0.04	79.77
TY12	8.58 ± 0.57	7.33 ± 0.76^a^	85.4
TY14	6.42 ± 0.74	3.57 ± 0.18	55.6
AM3	7.44 ± 0.62	5.5 ± 0.28	73.92
AM15	6.69 ± 0.46	4.23 ± 0.82	63.2
AM20	8.64 ± 0.45	6 ± 0.14	71
PR3	8.93 ± 0.26	5.46 ± 0.81	61
PR14	6.58 ± 0.79	5.64 ± 0.07	85.7
PR45	8.44 ± 0.14	5.45 ± 0.60	64.6
ER5	7.95 ± 0.09	6.11 ± 0.03^a^	76.8
MM39	7.78 ± 0.2	7.18 ± 0.2^a^	92.28
MSS10	8.43 ± 0.65	6.79 ± 0.48^a^	74.13
AB11	7.8 ± 0.30	6.6 ± 0.31^a^	84.61
C2	8.11 ± 0.04	3.93 ± 0.80	48.45
AB1	8.7 ± 0.2	6.86 ± 0.17^a^	80.3
AB8	7.52 ± 0.09	5.15 ± 0.02	68.48
W21	6.83 ± 0.08	3.54 ± 0.56	51.83

^1^Viable counts were transformed to log CFU/mL and expressed as mean ± SD from three experiments. ^a^Significant difference (*P* < 0.05) at log_10 _measurements after 12 h incubation at 0.3% compared to the initial count.

**Table 4:** Identification of oxalate degrading LAB strains by 16S rDNA sequences.

Isolates	GenBank accession number	The most matched organisms	Max identity (%)
TY5	KF588358	*L. fermentum* M059	99
TY12	KF588359	*L. fermentum* F-6	99
AM3	KF588357	*L. fermentum* F-6	100
ER5	KF588363	*W. confusa* K1-lb5	97
MSS10	KF588361	*W. cibaria* 4213	95
MM39	KF588362	*W. confusa* FS066	99
AB11	KF588360	*L. salivarius LB-33 *	99
AB8	KF588355	*L. fermentum* F-6, 6.1	99
AB1	KF588356	*L. fermentum IFO* 3956	99

**Table 5:** Antimicrobial activity of LAB isolates.

Isolates	*P. aeruginosa *	*B. subtilis *	*S. aureus *	*E. coli *	*B. cereus *	*S. typhi 25 *
TY5	+++	++	++	+	++	±
TY12	+++	++	++	+++	+++	−
AM3	+++	++	+++	+++	−	−
ER5	++	++	++	±	++	−
MM39	++	+	++	+	++	−
MSS10	++	++	+	−	+++	±
AB11	++	++	+++	++	+++	−
AB1	+	+	+	−	++	
AB8	+	+	−	++	++	−

− No inhibition, ± 0–4 mm, + 4–8 mm, ++ 8–12 mm, +++ ≥ 20 mm.

**Table 6:** Antibiotic susceptibility test of LAB isolates.

Isolates	Ampicillin	Chloramphenicol	Erythromycin	Tetracycline	Rifampicin	Kanamycin
TY5	R	S	S	S	R	R
TY12	I	S	S	I	R	R
AM3	R	S	I	I	R	R
ER5	R	I	R	R	R	R
MM39	R	S	I	R	R	R
MSS10	R	S	S	S	R	R
AB11	R	S	S	I	R	R
AB1	S	S	S	R	R	R
AB8	R	S	S	I	S	R

R: resistant; S: susceptible; I: intermediate.
